# Hyperglycemia and systemic inflammation differentially shape immune dysregulation, tissue destruction, and microbiota in experimental periodontitis and peri-implantitis in diabetic mice

**DOI:** 10.3389/fimmu.2026.1847456

**Published:** 2026-06-24

**Authors:** Takumi Memida, Jacques Christopher Jaar, Tsute Chen, Guoqin Cao, Nanako Kuriki, Elaheh Dalir Abdolahinia, Motoki Okamoto, Satoru Shindo, Shohei Yamashita, Xuesong He, Maiko Suzuki, Saynur Vardar, Toshihisa Kawai, Xiaozhe Han

**Affiliations:** 1 Department of Oral Science and Translational Research, College of Dental Medicine, Nova Southeastern University, Fort Lauderdale, FL, United States; 2 Department of Periodontal Medicine, Graduate School of Biomedical and Health Sciences, Hiroshima University, Hiroshima, Japan; 3 Department of Periodontology, College of Dental Medicine, Nova Southeastern University, Davie, FL, United States; 4 American Dental Association Forsyth Institute, Cambridge, MA, United States; 5 Department of Restorative Dentistry and Endodontology, Osaka University Graduate School of Dentistry, Osaka, Japan

**Keywords:** diabetes, hyperglycemia, microbiome, peri-implantitis, periodontitis, systemic inflammation

## Abstract

**Aim:**

To investigate the impact of hyperglycemia and systemic inflammation on experimental periodontitis/peri-implantitis in diabetic mice, focusing on osteoimmunological dysregulation and oral microbial alteration.

**Materials and methods:**

After implant placement, diabetic db/db mice were treated with Liraglutide, Indomethacin, or both, followed by ligature-induced experimental periodontitis/peri-implantitis. Samples were analyzed for bone loss, inflammatory cytokines, osteoclast activity, RAGE expression, IL-17-associated inflammatory responses, and Treg infiltration. The periodontal/peri-implant microbiota were examined by metagenomics and tested *in vitro* for inflammatory cytokine induction.

**Results:**

Liraglutide, but not indomethacin, effectively reduced bone loss, immune cell infiltration, RAGE, IL-17A expression, and restored Foxp3^+^ Treg presence. Post-treatment cytokine responses were slightly different between peri-implantitis sites compared to those in periodontitis sites. Oral microbiota composition from diabetic mice differed significantly from that of normoglycemic mice. Liraglutide treatment produced the greatest deviation from the ligation-only profile and shifted the microbiome toward normoglycemic control. The peri-implant microbiome was more resistant to interventions than the periodontal communities. Hyperglycemia control alleviated microbiome-induced pro-inflammatory responses *in vitro*.

**Conclusions:**

Diabetic hyperglycemia is a more predominant driver than systemic inflammation in exacerbating periodontitis/peri-implantitis tissue destruction, immune dysregulation, and eliciting a pro-inflammatory oral microbial environment. The local inflammatory response and microbial alteration around the tooth and implant were similar but not identical.

## 1 Introduction

Diabetes mellitus (DM) has emerged as a ubiquitous and prevalent metabolic disorder (1), and is associated with various complications (2). These complications are driven mainly by hyperglycemia and chronic low-grade systemic inflammation (3, 4). These metabolic and inflammatory conditions are closely linked to dysregulated immune responses.

Periodontitis has been considered the sixth complication of DM (5), with a bidirectional relationship worsening both conditions (6). Diabetic patients are also more susceptible than normoglycemic patients to peri-implantitis, an inflammatory disease affecting the soft and hard tissues surrounding dental implants (7), likely due to the impaired healing and heightened local inflammation (8). It is widely known that peri-implantitis presents a more accelerated and aggressive pattern of tissue destruction compared to periodontitis (9). However, the immunological basis underlying this increased susceptibility remains incompletely understood.

Oral dysbiosis is linked to increased virulence and impaired immune regulation in periodontitis and peri-implantitis (10–12). It has been associated with increased pathogenicity, impaired immune regulation, and heightened inflammatory signaling in patients with DM (13, 14).

Receptor for Advanced Glycation End-products (RAGE) mediates signaling pathways involved in inflammation and tissue destruction (15, 16). It has been reported that bacterial infection can indirectly enhance RAGE expression through the induction of proinflammatory cytokines (17). In addition, the imbalance of the Th17/regulatory T cell (Treg) has been elucidated to be involved in the progression of periodontitis and peri-implantitis (18, 19). In the DM condition, an increased Th17 differentiation and suppressed Treg function have been consistently reported, suggesting a shift toward a pro-inflammatory immune profile (20, 21). Together, these pathways highlight a potential link between metabolic stress and adaptive immune dysregulation.

The pathogenesis and molecular mechanisms by which DM contributes to local inflammation and microbial alteration are gradually being elucidated (22). A clinical study suggested that systemic inflammatory biomarkers were correlated with periodontitis (23). Our recent study has demonstrated a direct influence of elevated glucose levels on microbial alterations (24), consistent with findings that chronic hyperglycemia disrupts bone metabolism, enhances inflammatory cytokine production, and promotes alveolar bone loss through oxidative stress and AGE–RAGE signaling (25). These findings imply that both hyperglycemia and systemic inflammation may impair local immune regulation and contribute to the oral microbiome changes. However, whether these effects are primarily mediated through direct metabolic pathways or through immune dysregulation remains unclear.

Although hyperglycemia and systemic inflammation are tightly intertwined in diabetes and represent only part of its complex pathophysiology, hyperglycemia is widely regarded as an upstream driver of chronic inflammatory activation. The relative contributions of hyperglycemia and systemic inflammation to oral microbiota alterations and pathogenicity remain unclear, and few studies have directly compared their effects in periodontitis and peri-implantitis under controlled conditions. In particular, the extent to which hyperglycemia regulates local immune responses and host–microbiome interactions remains poorly defined.

Therefore, we hypothesized that hyperglycemia is the predominant systemic driver of immune dysregulation, tissue destruction, and oral microbiome alterations in experimental periodontitis/peri-implantitis under diabetic conditions, and peri-implant tissues are more susceptible than periodontal tissues to these hyperglycemia-associated changes. This study used a combined mouse model of experimental periodontitis and peri-implantitis to primarily quantify local tissue destruction using micro-computed tomography (micro-CT), as this outcome most directly reflects the pathological impact of hyperglycemia. To further elucidate the underlying immunological alteration, we additionally evaluated inflammatory cytokines, osteoclast activity, immune cell profiles, RAGE expression, and the composition and inflammatory potential of the oral microbiome.

## 2 Materials and methods

### 2.1 Animal

Four-week-old WT (C57BL/6J) and db/db (B6.BKS(D)-Leprdb/J) mice (Jackson Laboratory) were used. A total of 75 animals were used in this study. All mice were male. WT mice served as healthy controls. db/db mice were assigned to five groups (control, ligation, ligation + indomethacin, ligation + liraglutide, and combination). Sufficient implants were placed to obtain ≥8 successfully osseointegrated implants per group for quantitative analyses; thus, n represents animals with successful osseointegration. Indomethacin (Sigma-Aldrich) and liraglutide (Fisher Scientific) were administered to modulate systemic inflammation and hyperglycemia, respectively.

### 2.2 Tooth extraction, implantation, peri-implantitis induction, and peri-implant treatment

**Figure 1:**
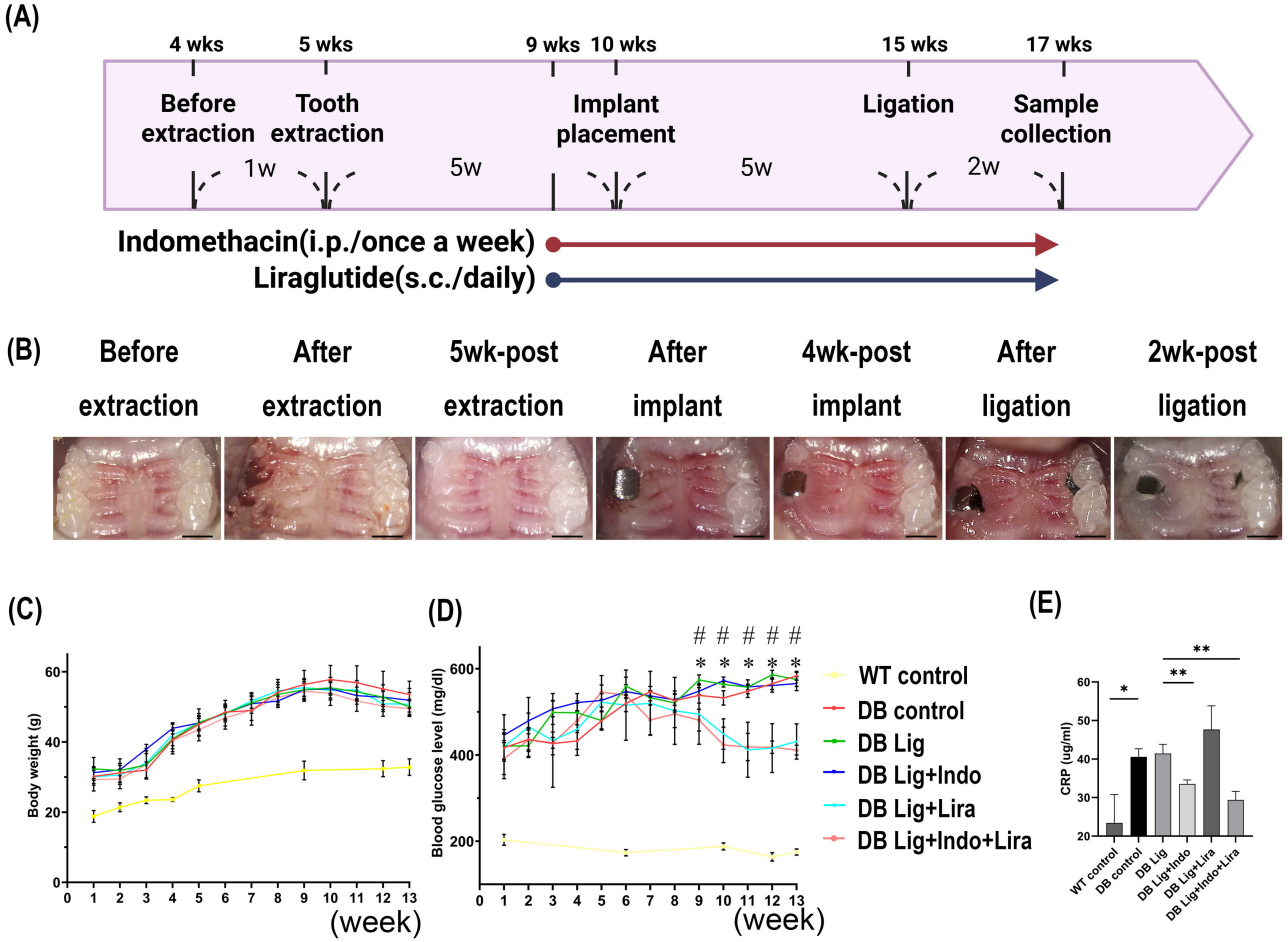
Experimental protocol and monitoring of body weight, blood glucose, and serum CRP levels. **(A)** Schematic illustration of the experimental schedule, including tooth extraction, implant placement, ligation, drug administration, and sample collection. Indomethacin was administered intraperitoneally once a week, and liraglutide was administered subcutaneously daily. **(B)** Representative clinical images showing the procedures of tooth extraction, implant placement, ligation, and post-ligation healing. Scale bar: 1 mm. **(C)** Body weight was monitored weekly. **(D)** Blood glucose levels were measured weekly. **(E)** Serum CRP levels were measured by ELISA at the endpoint. Data are shown as mean ± SEM (n = 6–7 per group). In panel **(D)** *p < 0.05 indicates DB Lig+Lira versus DB control, and #p < 0.05 indicates DB Lig+Indo+Lira versus DB control. In panel **(E)** *p < 0.05 and **p < 0.01 indicate the comparisons shown by horizontal lines. WT, wild-type; DB, diabetic; Lig, ligation around the tooth or implant; Indo, indomethacin; Lira, liraglutide.

Tooth extraction, implant placement, and ligature-induced peri-implantitis/periodontitis were performed as previously described (24). Each mouse received a titanium implant placed in the left maxillary edentulous ridge between the first and second molars, and the contralateral side (right maxillary second molar) was used to induce experimental periodontitis by 7–0 silk ligature placement. Indomethacin was prepared as a soluble formulation (1 mg/kg) and weekly administered intraperitoneally. Liraglutide was dissolved in sterile saline (400 μg/kg) and injected daily by subcutaneous injection into the dorsal neck region. The selected doses of liraglutide and indomethacin were based on previous murine studies demonstrating effective glycemic control and systemic anti-inflammatory effects, respectively, without significant toxicity (26–29). To minimize gastrointestinal toxicity associated with prolonged NSAID administration, a low-dose weekly indomethacin regimen was adopted. Both drugs were administered starting one week before implantation and continued until euthanasia. All surgical procedures were performed under inhalation anesthesia using isoflurane (induction at 3–4% and maintenance at 1–2%). At the experimental endpoint, mice were euthanized by CO_2_ inhalation. Surgical schedules are shown in **Figures 1A, B**.

### 2.3 Body weight measurement, blood glucose detection, and CRP expression

Body weight and random blood glucose were monitored weekly in both WT and db/db mice using a OneTouch glucometer (LifeScan). Blood glucose was measured from the tail vein (10 µL). Serum CRP concentrations were assessed by ELISA (R&D Systems) at the experimental endpoint according to the manufacturer’s instructions.

### 2.4 Micro-computed tomography scanning and bone loss volume analysis

Maxillary samples consisted of the alveolar bone and surrounding gingival tissues harvested from the mesial of the first molar to the distal of the third molar region, including the implant or ligated second molar site as appropriate. Maxillae were fixed in 4% paraformaldehyde and scanned using a high-resolution micro-CT system (Skyscan), following established protocols (24). Using a split-mouth design, peri-implant and periodontal bone loss were assessed at the maxillary implant site and contralateral ligated second molar, respectively, and quantified as averaged mesial and distal interproximal volumetric defects by micro-CT. Standardized volumes of interest (VOIs) were defined around the implants and second molars while excluding the implant or tooth structure. Bone loss was quantified as empty space volume (ESV = total volume − bone volume) using CTAn software.

### 2.5 Hematoxylin and eosin staining

Maxillae were decalcified in 10% EDTA at 4 °C for three weeks, embedded in paraffin, and sectioned longitudinally at 5 µm. Sections were stained with hematoxylin and eosin using standard protocols. (30). Inflammatory cell infiltration was quantified in four standardized regions (400 µm × 400 µm) within peri-implant or periodontal connective tissue using ImageJ, and the mean value per sample was calculated.

### 2.6 Tartrate-resistant acid phosphatase staining

Decalcified, paraffin-embedded maxillary specimens were sectioned longitudinally at 5 µm and stained for TRAP using standard protocols (30). Multinucleated TRAP-positive osteoclasts (≥3 nuclei) along the alveolar bone surface were counted in standardized mesial and distal peri-implant and periodontal regions under 40× magnification. The mean osteoclast number per sample was calculated.

### 2.7 Immunohistochemistry staining

Paraffin-embedded maxillary sections were deparaffinized and subjected to antigen retrieval in citrate buffer (pH 6.0). After blocking, sections were incubated overnight with primary antibodies against IL-17A and RAGE (1:200, Abcam). HRP-conjugated secondary antibodies (VECTASTAIN^®^ ABC kit, Vector Laboratories) and ImmPACT^®^ VIP substrate were used for signal detection, followed by hematoxylin counterstaining. Positive cells were quantified using ImageJ within standardized high-power fields, and the mean value per sample was calculated.

### 2.8 Immunofluorescence staining

Paraffin-embedded maxillary sections were deparaffinized and subjected to antigen retrieval in Tris-EDTA buffer (pH 9.0). After blocking, sections were incubated overnight with APC-conjugated anti-CD4 (1:100, BioLegend) and rabbit anti-FOXP3 (1:100, Abcam). Alexa Fluor 488-conjugated anti-rabbit IgG (1:500, Invitrogen) was used as the secondary antibody, and nuclei were counterstained with DAPI (1:1000, Invitrogen). Fluorescence images were acquired using an EVOS M5000 system. FOXP3^+^CD4^+^ double-positive cells were quantified using ImageJ within standardized high-power fields.

### 2.9 Quantitative RT-PCR

Total RNA was extracted from the gingival tissues at the implant and tooth sites and reverse-transcribed into cDNA. Quantitative PCR was performed using SYBR Green Master Mix with gene-specific primers. Gene expression levels were normalized to Gapdh and calculated using the ΔΔCt method. Primer sequences are provided in **Supplementary Table 1**.

### 2.10 Bacteria isolation and expansion

Biofilm-associated microbiota were collected from silk ligatures or oral swabs as previously described (24). At the endpoint, biofilm-associated microbiota were collected from silk ligatures in ligature groups, whereas non-ligated periodontal or peri-implant sites were gently sampled using oral swabs. Each sample was divided for 16S rRNA sequencing or for bacterial expansion according to established culture protocols (31). Expanded microbiota were subsequently used for co-culture assays with primary murine splenocytes.

### 2.11 Co-culture of bacteria with splenocytes

Primary splenocytes were isolated from C57BL/6J mice as previously described (32). Aerobic or anaerobic bacterial suspensions were fixed in 4% paraformaldehyde, washed with PBS, and adjusted in concentration based on OD_600_. Fixed bacteria were co-cultured with splenocytes at a 1:500 ratio for 12 h at 37 °C. After incubation, total RNA was extracted from splenocytes using a commercial RNA extraction kit (Invitrogen).

### 2.12 16S rRNA gene sequencing and sample processing

All samples were collected at the experimental endpoint immediately prior to sacrifice and snap-frozen at −80 °C until DNA extraction using the DNeasy^®^ PowerSoil^®^ Pro Kit (QIAGEN). The V3–V4 region of the 16S rRNA gene was sequenced on the Illumina MiSeq platform (2 × 300 bp, CD Genomics). Raw reads were processed with DADA2 for quality control and ASV inference, and diversity and taxonomic analyses were performed using QIIME2 (v2025.4) (33).

### 2.13 Statistical analysis

Statistical analyses were performed using GraphPad Prism v10. One-way ANOVA followed by Dunnett’s *post hoc* test was used when comparing multiple treatment groups with a single control, whereas Tukey’s *post hoc* test was applied for all pairwise comparisons. A p-value < 0.05 was considered statistically significant.

## 3 Results

### 3.1 Liraglutide and indomethacin selectively modulate blood glucose and CRP

The success rate of implant osseointegration was higher in WT mice (80%, 8/10 implants) compared with that in the db/db mice group (52.3%, 34/65 implants). To confirm treatment efficacy, we assessed body weight, blood glucose, and CRP levels. Body weight progressively increased in all db/db groups regardless of treatment (**Figure 1C**). Liraglutide significantly reduced elevated blood glucose levels from 9 weeks to the end as compared to the Ligation group (**Figure 1D**), while indomethacin significantly decreased serum CRP levels at endpoint as compared to the Ligation group (p<0.01, **Figure 1E**).

### 3.2 Hyperglycemia plays a dominant role in bone loss and osteoclastogenesis induction at periodontal and peri-implant sites

**Figure 2:**
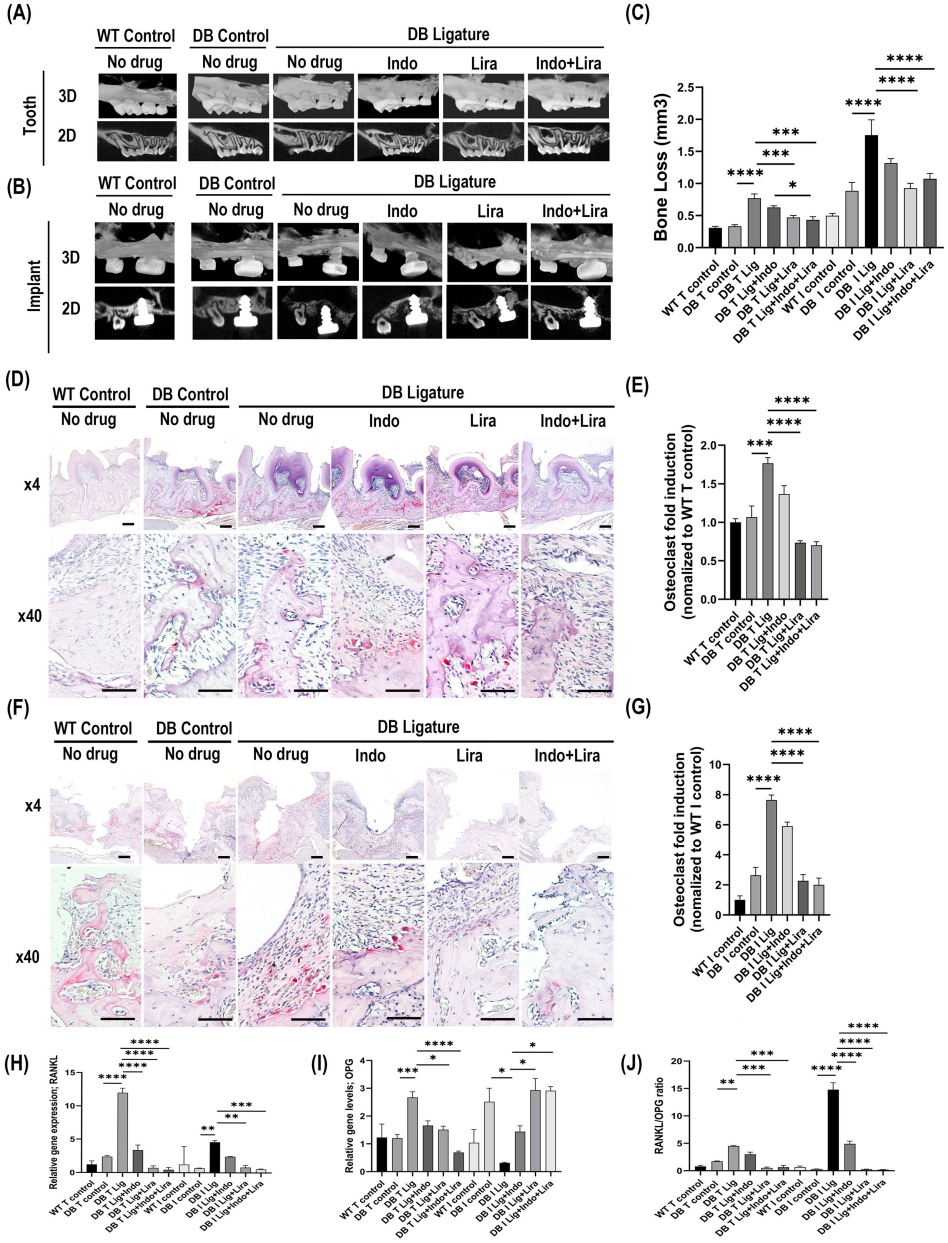
Evaluation of alveolar bone resorption, osteoclast formation, and RANKL/OPG balance in periodontal and peri-implant tissues. **(A)** Representative three-dimensional and two-dimensional sagittal micro-CT images of periodontal tissues around teeth. **(B)** Representative three-dimensional and two-dimensional sagittal micro-CT images of peri-implant tissues around implants. **(C)** Quantification of bone loss volume around teeth and implants (n = 6–7 per group). **(D)** Representative TRAP-stained images of periodontal tissues around teeth. Scale bar: 150 µm. **(E)** TRAP-positive osteoclasts around teeth were counted and normalized to the WT control group (n = 3 per group). **(F)** Representative TRAP-stained images of peri-implant tissues around implants. Scale bar: 150 µm. **(G)** TRAP-positive osteoclasts around implants were counted and normalized to the WT control group (n = 3 per group). Gingival mRNA expression levels of **(H)** RANKL and **(I)** OPG were measured by qPCR, and **(J)** the RANKL/OPG ratio was calculated based on their mRNA expression levels (n = 3–4 per group). Data are shown as mean ± SEM. *p < 0.05, **p < 0.01, ***p < 0.001, ****p < 0.0001. WT, wild-type; DB, diabetic; T, tooth; I, implant; Lig, ligation around the tooth or implant; Indo, indomethacin; Lira, liraglutide.

Micro-CT analysis revealed significant bone loss at both tooth- and implant-associated sites following ligation (p<0.0001, **Figures 2A–C**). Bone loss was significantly reduced by liraglutide or combined treatment (p<0.0001) compared to the ligation group, whereas indomethacin alone had no effect. At tooth sites, bone loss was significantly lower in the combined treatment group than in the indomethacin group (p<0.05), whereas no significant difference was observed between the treatments at implant sites. Although both sites showed similar trends, peri-implant bone loss was significantly greater than periodontal bone loss (p<0.0001).

Moreover, TRAP staining showed elevated osteoclast numbers in ligated tissues, which were significantly reduced by liraglutide and combination treatments as compared to the ligation group, but not by indomethacin (p<0.001, **Figures 2D–G**). Notably, osteoclast formation was more pronounced at peri-implant sites than at periodontal sites. *Rankl* expression was significantly increased after ligation at both sites, with greater expression on the tooth side (p<0.0001; **Figure 2H**). This increase was significantly suppressed by liraglutide or combination treatment, but only partially reduced by indomethacin on the implant side, as compared to the ligation group. *Opg* expression was upregulated on the tooth side and downregulated on the implant side in the Ligation group (**Figure 2I**) following liraglutide or combination treatment, but not indomethacin treatment, as compared to the ligation group (p<0.05). Consequently, the *Rankl*/*Opg* ratio remained significantly higher at peri-implant sites than at periodontal sites when compared to the ligation group (p<0.0001; **Figure 2J**).

### 3.3 Hyperglycemia strongly disrupts the gingival cytokine balance

**Figure 3:**
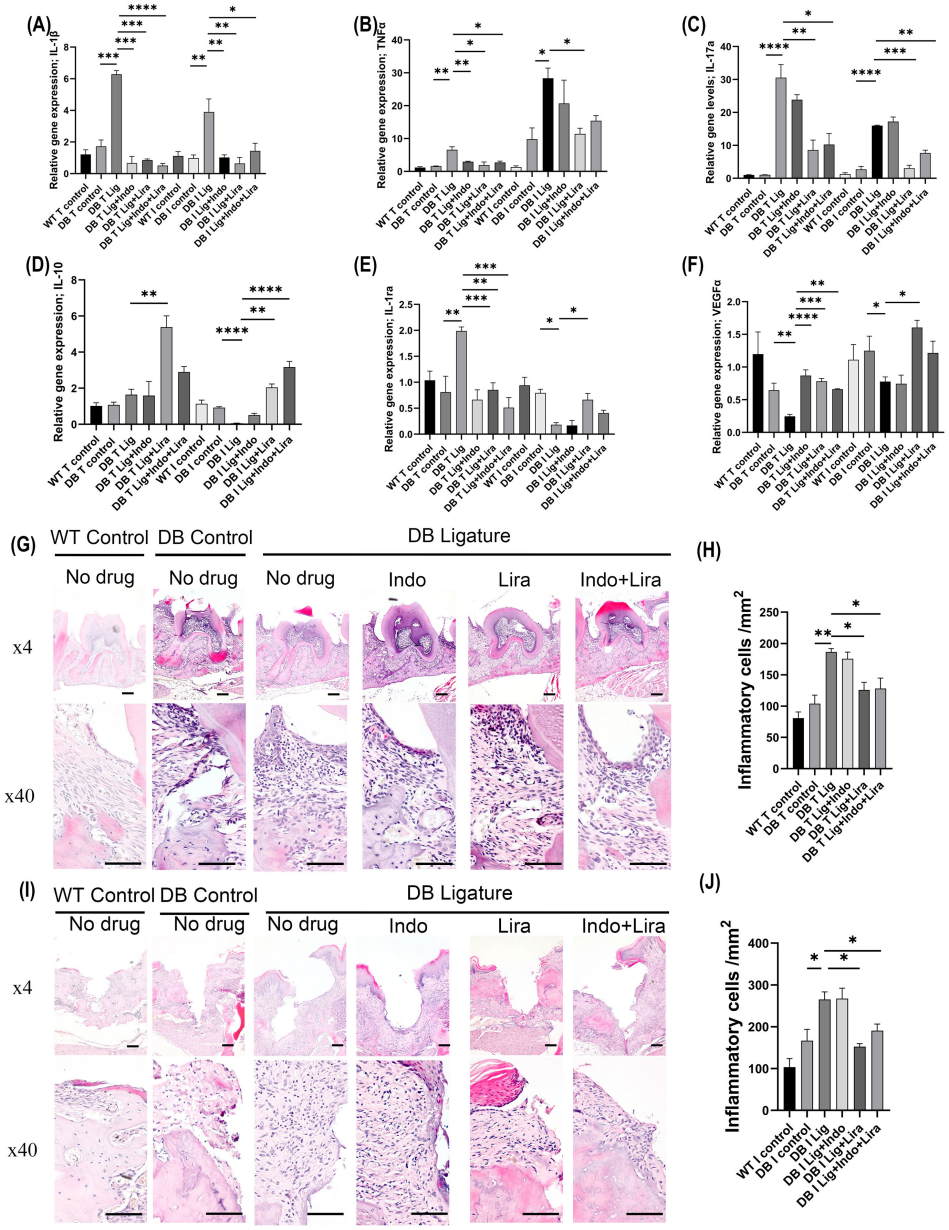
Gingival mRNA expression of inflammatory and regulatory mediators and histological evaluation of inflammatory cell infiltration. Gingival mRNA expression levels of **(A)** IL-1β, **(B)** TNF-α, **(C)** IL-17a, **(D)** IL-10, **(E)** IL-1ra, and **(F)** VEGF-α were measured by qPCR (n = 3–4 per group). **(G)** Representative HE-stained images of periodontal tissues around teeth. Scale bar: 150 µm. **(H)** Inflammatory cells around teeth were counted using ImageJ (n = 3 per group). **(I)** Representative HE-stained images of peri-implant tissues around implants. Scale bar: 150 µm. **(J)** Inflammatory cells around implants were counted using ImageJ (n = 3 per group). Data are shown as mean ± SEM. *p < 0.05, **p < 0.01, ***p < 0.001, ****p < 0.0001. WT, wild-type; DB, diabetic; T, tooth; I, implant; Lig, ligation around the tooth or implant; Indo, indomethacin; Lira, liraglutide.

*Il1b*, *Tnfa*, and *Il17a* expression levels were significantly increased in the ligation group at both periodontal and peri-implant sites compared to controls (p<0.05; **Figures 3A–C**). Expression of these pro-inflammatory cytokines was effectively suppressed by liraglutide or combination treatment (p<0.05), while indomethacin alone only showed suppression of *Ill1b* and *Tnfa* on the tooth side (**Figures 3A, B**), but not on the implant side. *Il17a* expression was significantly reduced only in the liraglutide-treated groups compared to the control group (**Figure 3C**). *Il10* and *Il1ra* expression levels were consistently higher in periodontal tissues than in peri-implant tissues (p<0.01; **Figures 3D, E**). *Il10* expression was significantly increased in the liraglutide-treated group on the tooth side, and in both the liraglutide and combination group on the implant side, compared to the control group (p<0.01; **Figure 3D**). In contrast, *Il1ra* expression, which was elevated on the tooth side in the ligation group, was decreased significantly following all treatments (p < 0.01). On the implant side, however, expression was markedly lower in the ligation group than in the controls, and recovery occurred only in the liraglutide group (p<0.05; **Figure 3E**). *Vegfa* expression, which was downregulated by ligation on both sides (p<0.05), was more effectively restored in liraglutide group and combination group, particularly on the implant side (p<0.05; **Figure 3F**).

### 3.4 Glycemic control effectively reduces immune cell infiltration

HE staining revealed increased immune cell infiltration in gingival tissues at both periodontal and peri-implant sites following ligation compared to controls (p<0.05; **Figures 3G–J**), which tended to be more prominent at peri-implant sites. Liraglutide and combination treatment significantly reduced this inflammatory infiltration at both sites (p<0.05), while indomethacin alone showed only limited effects compared to the ligation group.

### 3.5 Hyperglycemia strongly induced RAGE expression in the gingival tissues

**Figure 4:**
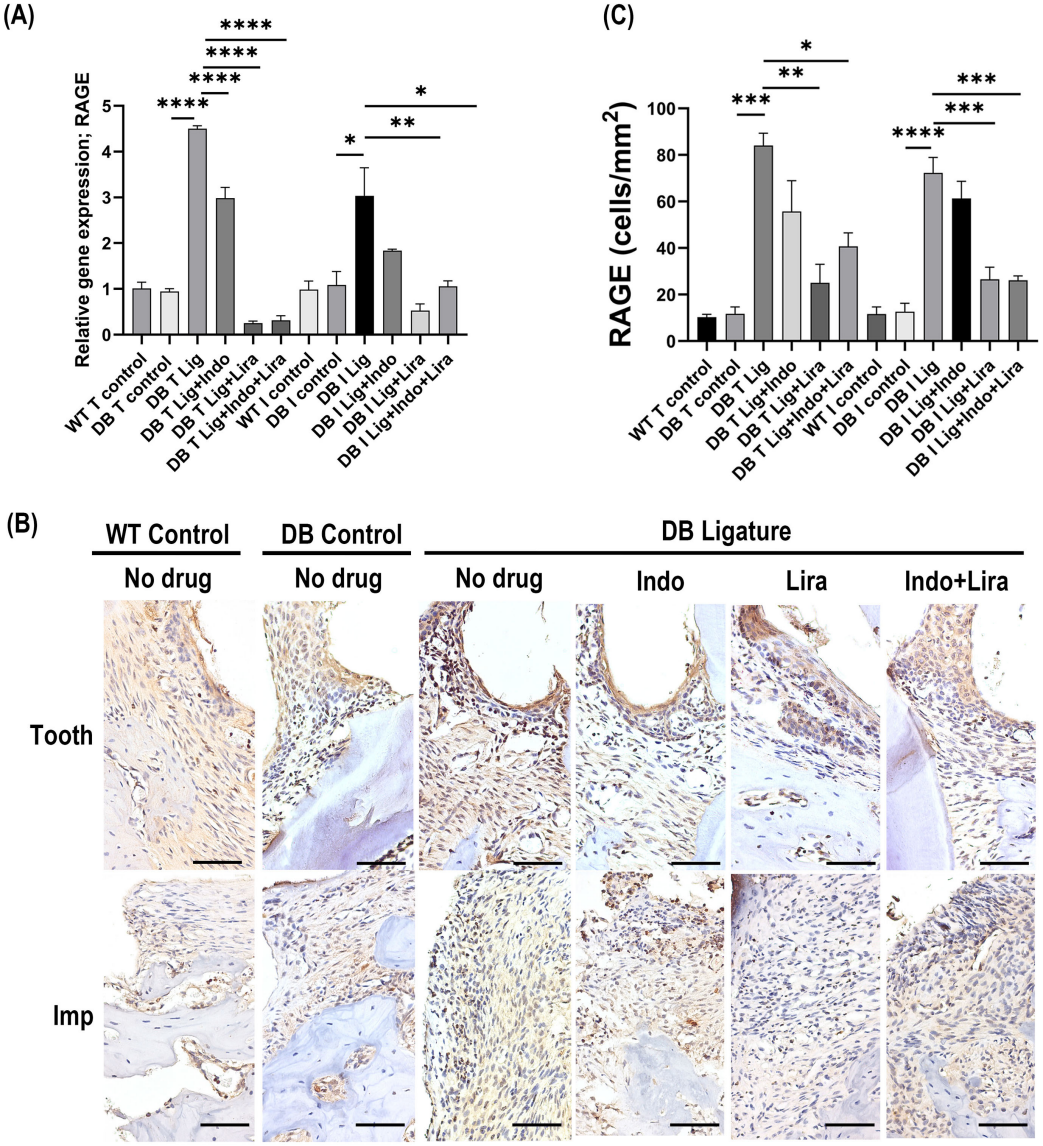
Detection of RAGE expression in periodontal and peri-implant tissues at mRNA and protein levels. **(A)** Gingival mRNA expression levels of RAGE were measured by qPCR (n = 4 per group). **(B)** Representative IHC images of RAGE expression in periodontal and peri-implant tissues. Scale bar: 150 µm. **(C)** RAGE-positive cells were quantified as cells/mm² using ImageJ (n = 3 per group). Data are shown as mean ± SEM. *p < 0.05, **p < 0.01, ***p < 0.001, ****p < 0.0001. WT, wild-type; DB, diabetic; T, tooth; I, implant; Lig, ligation around the tooth or implant; Indo, indomethacin; Lira, liraglutide.

Receptor for Advanced Glycation End-products (RAGE) expression was significantly upregulated by ligation at both periodontal and peri-implant sites compared to controls (p<0.05; **Figures 4A–C**). This upregulation was markedly suppressed by liraglutide and combination treatment (p<0.0001), while indomethacin alone showed limited effects compared to the ligation group. IHC analysis supported these findings, revealing a significant reduction in RAGE-positive cells in the liraglutide and combination groups (p<0.05), but not in the indomethacin group at either site compared to the ligation group.

### 3.6 Hyperglycemia predominantly alters local immune regulation

**Figure 5:**
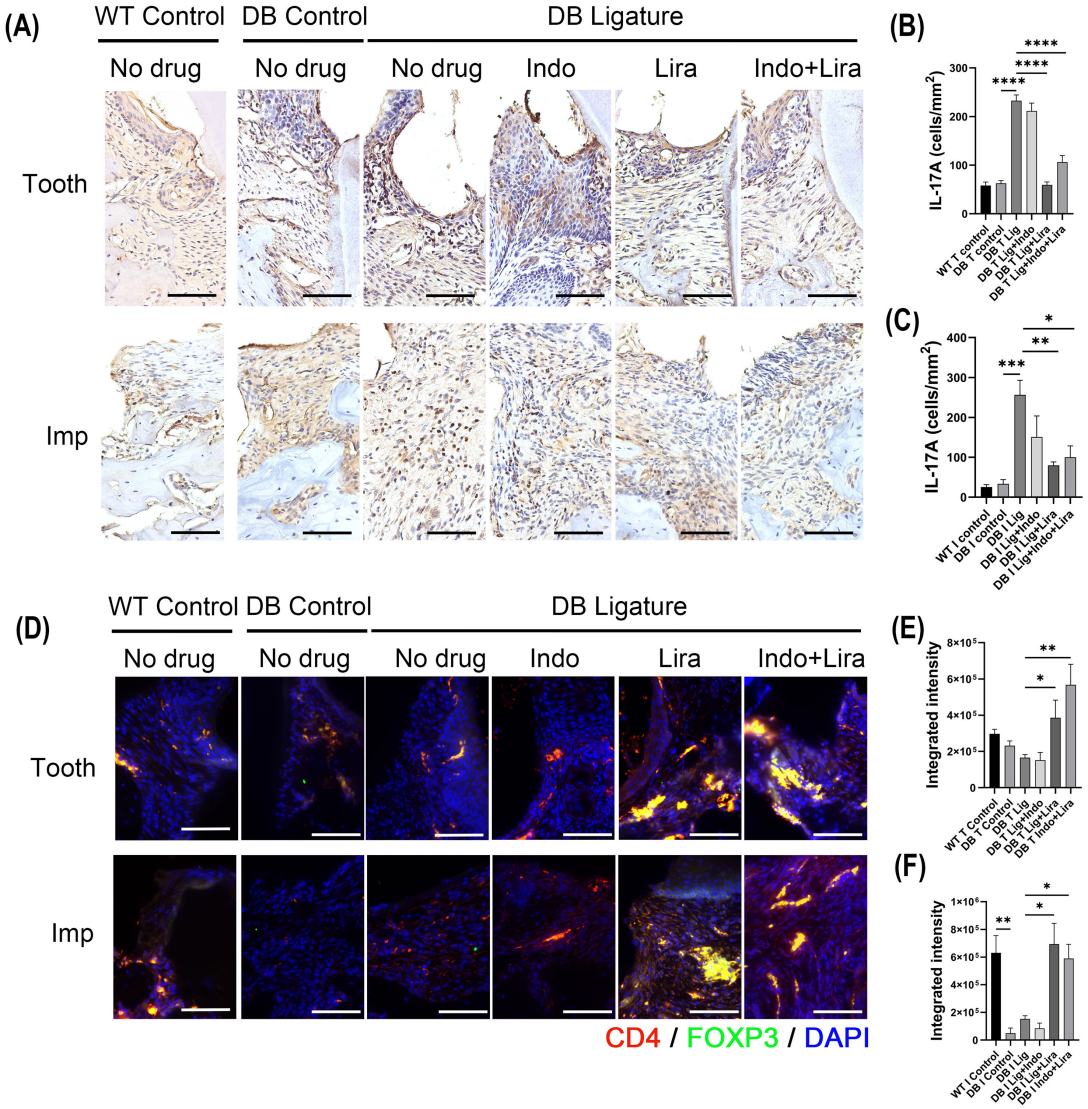
Detection of IL-17A expression and CD4/FOXP3 signals in periodontal and peri-implant tissues. **(A)** Representative IHC images of IL-17A expression in periodontal and peri-implant tissues. Scale bar: 150 µm. **(B, C)** IL-17A-positive cells were quantified as cells/mm² in periodontal tissues around teeth **(B)** and peri-implant tissues around implants **(C)** using ImageJ (n = 3 per group). **(D)** Representative immunofluorescence images of CD4, FOXP3, and DAPI staining in periodontal and peri-implant tissues. CD4, FOXP3, and nuclei are shown in red, green, and blue, respectively. Scale bar: 150 µm. **(E, F)** Integrated fluorescence intensity of CD4/FOXP3 signals was quantified in periodontal tissues around teeth **(E)** and peri-implant tissues around implants **(F)** using ImageJ (n = 3 per group). Data are shown as mean ± SEM. *p < 0.05, **p < 0.01, ***p < 0.001, ****p < 0.0001. WT, wild-type; DB, diabetic; T, tooth; I, implant; Lig, ligation around the tooth or implant; Indo, indomethacin; Lira, liraglutide.

To investigate the influence of systemic factors on local immune regulation, IL-17A, CD4, and FOXP3 expression in gingival tissues were assessed by IHC and IF staining. IL-17A expression was significantly elevated in ligated tissues at both periodontal and peri-implant sites, and was markedly reduced by liraglutide treatment, but not by indomethacin, compared to the ligation group (p<0.05; **Figures 5A–C**). Furthermore, CD4^+^FOXP3^+^ regulatory T cells were minimally present in the ligation group at either site. Notably, even under control conditions at the implant sites, Treg cell infiltration was markedly reduced in db/db mice compared to WT controls. In contrast, Treg recruitment was significantly restored in the liraglutide and combination treatment groups, but not in the indomethacin group (p<0.05; **Figures 5D–F**).

### 3.7 Hyperglycemia control more effectively ameliorated oral microbial alteration and reduced inflammatory responses by splenocytes *in vitro*

**Figure 6:**
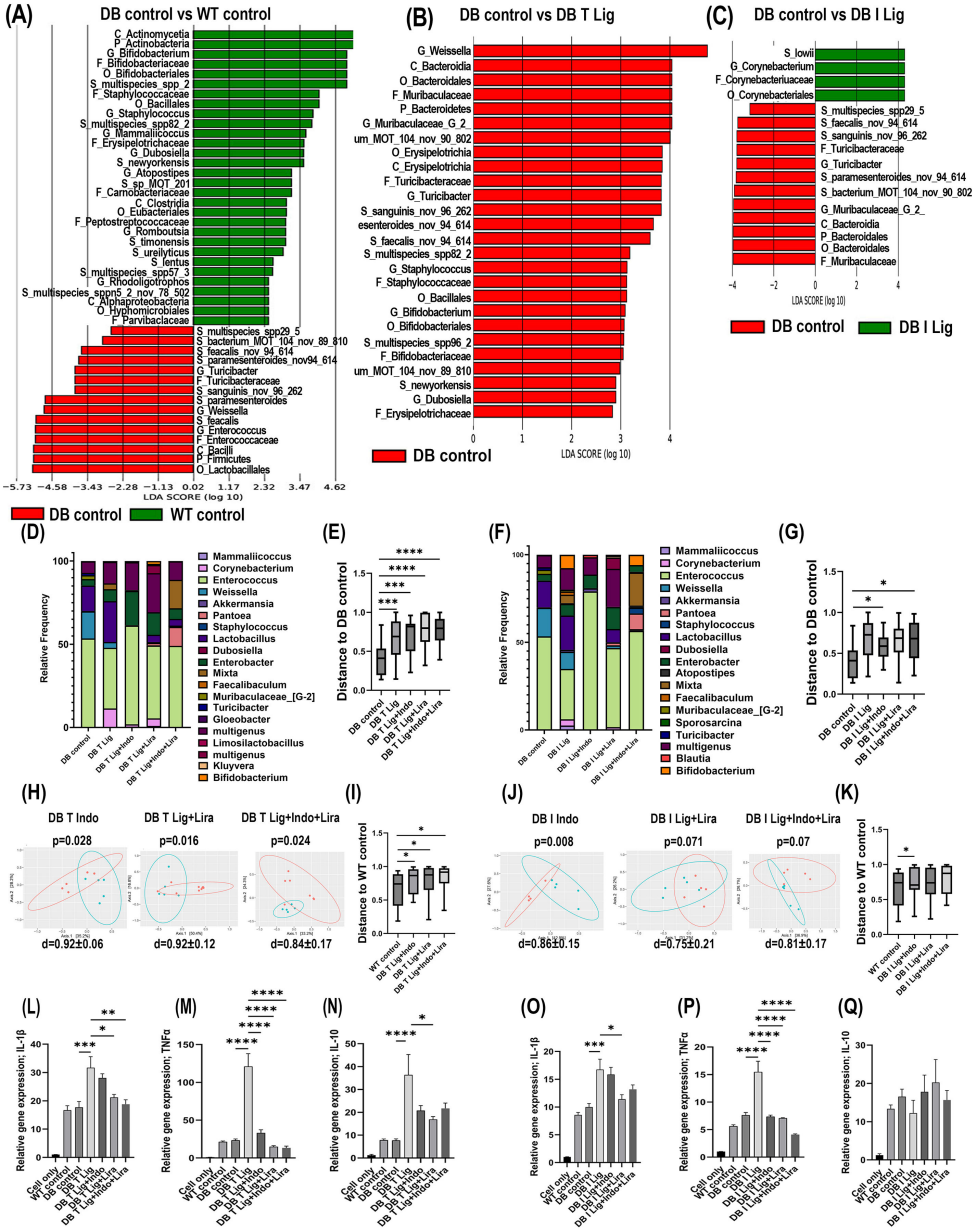
Microbiome composition and pro-inflammatory potential of oral microbiota from periodontal and peri-implant sites in diabetic and WT mice. **(A–C)** Linear discriminant analysis effect size (LEfSe) identifying taxa differentially abundant between groups: DB control versus WT control **(A)**, DB control versus DB tooth ligation group (T Lig) **(B)**, and DB control versus DB implant ligation group (I Lig) **(C)**. Red and green bars indicate taxa enriched in the corresponding groups shown in each panel. **(D, F)** Relative abundance of dominant bacterial genera in periodontal **(D)** and peri-implant **(F)** sites across treatment groups. **(E, G)** Bray–Curtis distances from DB control microbiota in periodontal **(E)** and peri-implant **(G)** sites. Boxplots show the median, interquartile range, and range. **(H, J)** Principal coordinate analysis (PCoA) plots based on Bray–Curtis distances of periodontal **(H)** and peri-implant **(J)** microbiota, showing clustering by treatment group. PERMANOVA p-values are indicated in the plots. The indicated d values represent Bray–Curtis distances between compared groups. **(I, K)** Bray–Curtis distances from WT control microbiota in periodontal **(I)** and peri-implant **(K)** sites. Statistical significance for beta-diversity analyses was assessed by PERMANOVA. **(L–Q)** Relative mRNA expression levels of IL-1β, TNF-α, and IL-10 in splenocytes co-cultured with oral microbiota isolated from periodontal sites **(L–N)** or peri-implant sites **(O–Q)**. Data are shown as mean ± SEM for bar graphs and as boxplots for beta-diversity distance analyses (n = 4–6 per group for microbiome analyses; n = 6 per group for cell culture assays). *p < 0.05, **p < 0.01, ***p < 0.001, ****p < 0.0001. WT, wild-type; DB, diabetic; T, tooth; I, implant; Lig, ligation; Indo, indomethacin; Lira, liraglutide.

The 16S rRNA sequencing analysis was performed on microbiota isolated from ligatures around teeth and implants. Clear compositional differences were observed between WT and DB controls (**Figure 6A**). Relative to DB controls, the relative abundance of microbial species in the ligation group was reduced, with a more pronounced effect at tooth sites than implant sites (**Figures 6B, C**). This trend was consistent with the α-diversity results (**Supplementary Figures 1A, B**), which showed significant differences from the DB control group at tooth sites but not at implant sites. Taxonomic analysis at the genus level revealed compositional changes in the oral microbiota at both tooth and implant sites following intervention (**Figures 6D, F**). In β-diversity analysis, pairwise comparisons with DB controls showed significant distances for all intervention groups at tooth sites, and for the indomethacin-treated group at implant sites (**Figures 6E, G**). However, no significant differences were detected among the intervention groups. The β-diversity analysis relative to WT showed that, on the tooth side, all treatment groups remained significantly different from WT controls (**Figures 6H, I**). In contrast, on the implant side, only the indomethacin group differed significantly from WT controls, whereas the liraglutide and combined groups showed no significant difference (**Figures 6J, K**).

To evaluate functional pathogenicity, the site-specific microbiome isolated from each condition was expanded and co-cultured with splenocytes. At both sites, ligation-derived microbiota increased IL-1β and TNF-α expression relative to controls, and this up-regulation was attenuated by liraglutide and by the combined treatment in splenocytes (p<0.05; **Figures 6L, M, O, P**). IL-10 expression was increased on the tooth side in the ligation group (p<0.05; **Figures 6N, Q**), but not on the implant side. After interventions, IL-10 expression was reduced by liraglutide treatment compared to the ligation group, whereas no significant differences were observed at implant sites.

## 4 Discussion

This study provides insight into how hyperglycemia may contribute to immune dysregulation and tissue destruction in experimental periodontitis and peri-implantitis. Our *in vivo* data indicate that glycemic control attenuated alveolar bone loss, immune cell infiltration, osteoclast activity, and *Rankl/Opg* imbalance at both periodontal and peri-implant sites, whereas control of systemic inflammation alone showed more limited effects (**Figures 1**, **2**). These findings support a stronger contribution of hyperglycemia, compared with systemic inflammation, to peri-implant tissue destruction in diabetes, consistent with clinical evidence showing increased peri-implantitis risk in hyperglycemic patients (34) and our previous experimental study (24). Importantly, these effects may be associated with immune dysregulation, suggesting that hyperglycemia may influence osteoimmunological imbalance rather than solely enhancing inflammatory output.

Across all parameters evaluated, including bone loss (**Figures 1A–C**), osteoclast number (**Figures 1D–G**), inflammatory cytokine imbalances (**Figures 2A–F**), and RAGE activation (**Figures 4A–C**), the peri-implant site generally exhibited more pronounced inflammatory responses and tissue destruction compared with the periodontal site. PDL-derived cells secrete protective cytokines such as *Il10*, *Tgfb*, *Il1ra*, and *Opg*, which play crucial roles in immune regulation and inhibition of osteoclastogenesis (35). Moreover, previous studies have suggested that titanium particles released from implants may exacerbate local immune responses in peri-implant tissues (36). These structural and material-related features may contribute to the elevated susceptibility of peri-implant tissues to immune dysregulation and inflammatory bone loss, although further studies are required to clarify the underlying mechanisms.

A dissociation was observed between bone loss and osteoclast number or *Rankl/Opg* expression (**Figure 2**), which is not unexpected in inflammatory peri-implantitis models. Micro-CT reflects cumulative bone loss, whereas osteoclast counts and gene expression represent terminal time-point measures, and inflammatory bone resorption can also occur through RANKL-independent mechanisms. In diabetic conditions, this dissociation may reflect sustained immune activation and impaired resolution pathways, which could contribute to tissue destruction beyond osteoclast-mediated mechanisms, as reported in previous studies (25, 37, 38).

Cytokine profiling showed increased pro-inflammatory cytokines (*Ill1b*, *Tnfa*, *Il17a*) after ligation at both tooth and implant sites, whereas anti-inflammatory cytokines (*Il10*, *Il1ra*) and *Opg* expression were suppressed in peri-implant tissues, indicating a site-specific susceptibility to immune dysregulation (**Figure 2I**, **3A-F**). This imbalance was more effectively improved by liraglutide than by indomethacin. Structural and immunological differences between periodontal and peri-implant tissues, including reduced vascular supply and absence of the periodontal ligament, may impair inflammatory resolution at implant sites (39), consistent with the higher susceptibility to peri-implant tissue destruction reported in clinical studies (40). Foxp3^+^ Tregs were better preserved in the liraglutide group, whereas limited changes were observed with indomethacin (**Figure 5**), suggesting a role of hyperglycemia in IL-17-associated immune dysregulation and Treg responses in peri-implantitis (19, 41).

RAGE expression was elevated under hyperglycemia and reduced by liraglutide (**Figure 4**). AGE–RAGE signaling is known to promote NF-κB–dependent inflammation and oxidative stress (42, 43). In this study, increased *Rage* coincided with higher *Il1b*, *Tnfa*, and *Il17a* and lower *Il10* and *Il1ra*, particularly at implant sites, suggesting that AGE–RAGE signaling may contribute to a sustained local inflammation. The lack of RAGE suppression with indomethacin may be related to its limited local immunomodulatory effect. Causality cannot be established from these data and requires further investigation.

Our results suggest that both hyperglycemia and systemic inflammation influence oral microbiota composition and pathogenic potential in diabetes, with site-specific responses to intervention. β-diversity differed from DM controls across treatments, indicating detectable community shifts, whereas α-diversity was reduced only at periodontal sites, suggesting greater richness loss than at peri-implant sites. The relative stability of peri-implant diversity may reflect a pre-existing dysbiotic state under diabetic conditions, consistent with clinical reports showing limited effects of diabetes on peri-implant submucosal microbiota compared with periodontal sites (44). Importantly, stable diversity did not necessarily correlate with reduced pro-inflammatory potential, as peri-implant microbiota induced stronger *Il1b* and *Tnfa* responses in splenocyte cultures and showed limited *Il10* induction, suggesting site-dependent differences in functional pathogenicity. This may reflect differences in the pathogenicity of these microbial communities, possibly influenced by local tissue structure and immune microenvironments. These observations support the concept of bidirectional host–microbiome interactions, in which immune status influences microbial function. Although evidence remains limited, meta-transcriptomic findings suggest that peri-implant infections are primarily driven by the functional pathogenicity of their microbial communities, which is not fully captured by taxonomic composition alone (45, 46). At peri-implant sites, liraglutide and combination treatments shifted microbial communities toward WT-like profiles, whereas indomethacin-treated groups remained distinct. These findings suggest that metabolic modulation may more effectively influence peri-implant microbial communities, potentially through suppression of AGE–RAGE signaling (47) and immune regulation (48). However, indomethacin, which does not improve glycemic status, showed limited effects on microbial and immune alterations (49). Overall, these results indicate that hyperglycemia plays a major role in exacerbating and sustaining potential pathological alteration of the oral microbiome in DM conditions, and that glycemic control is effective in shifting peri-implant microbial communities toward a less harmful state. However, interpretation of murine oral microbiome data remains limited because disease-defining oral pathogens and dysbiotic signatures are less clearly established in mice than in humans. Therefore, the microbial alterations observed in this study should primarily be interpreted as compositional shifts associated with diabetic and inflammatory conditions rather than definitive causal drivers of tissue destruction.

Liraglutide was used to control hyperglycemia in db/db mice, whereas indomethacin was administered as a systemic anti-inflammatory agent to reduce the background inflammatory burden partially. Combination therapy showed no clear advantage over liraglutide alone, supporting a predominant role of hyperglycemia in this model. Persistent hyperglycemia is linked to sustained inflammation and impaired insulin sensitivity, which may limit the efficacy of anti-inflammatory treatment alone (50). In addition, liraglutide may exert anti-inflammatory effects independent of glycemic control, including modulation of macrophage inflammatory responses, as suggested in previous studies. Therefore, some of the beneficial effects observed in the current study may not be attributable solely to glucose lowering.

This study has some limitations. Although the db/db mouse is a well-established model of type 2 diabetes, species-specific differences in oral anatomy, immune responses, and microbiota composition should be considered when extrapolating the findings to humans. In addition, microbiome profiling was based on 16S rRNA gene sequencing, and future studies incorporating functional analyses may further clarify the roles of dysbiotic microbiota. The mechanistic interpretation of immune dysregulation also remains limited. Although IL-17A-associated inflammatory responses and CD4^+^FOXP3^+^ cells were evaluated histologically to preserve local tissue context, flow cytometric analysis would be required to more precisely define Th17 and Treg populations. Similarly, RAGE expression was evaluated as one of the diabetes-associated tissue markers under inflammatory conditions, but AGE accumulation and the direct functional role of RAGE were not examined. Further studies are needed to clarify how these immune and diabetes-associated pathways directly contribute to local inflammation and tissue destruction in diabetic periodontitis and peri-implantitis.

Peri-implant tissues consistently exhibited more severe inflammatory and osteoclastic changes than periodontal tissues, suggesting a heightened susceptibility in the peri-implant environment. These results support current consensus recommendations to improve implant outcomes in diabetic patients, including documenting preoperative HbA1c within 3 months (goal ≤7%), deferring implant surgery when control is poor (HbA1c ≥8–9%), and scheduling short-interval maintenance with continued glycemic monitoring (51, 52). Moreover, current clinical guidelines emphasize that mechanical biofilm control is the cornerstone of periodontal and peri-implant therapy, with systemic risk factor management—particularly glycemic control in patients with diabetes—serving as an important adjunct. Consistent with this framework, our findings show that improving hyperglycemia resulted in broader improvements in tissue destruction and inflammatory dysregulation in a biofilm-driven model, whereas systemic anti-inflammatory treatment alone had more limited effects. Importantly, the systemic pharmacologic interventions used in this experimental study are not intended to replace standard clinical biofilm control measures, but rather to help dissect the relative contributions of hyperglycemia and systemic inflammation under conditions of persistent microbial challenge (53, 54).

Overall, our findings suggest that hyperglycemia may be an important contributor to immune dysregulation, local inflammation, bone loss, and microbiota pathogenicity in diabetic periodontitis and peri-implantitis. Furthermore, periodontal and peri-implant inflammatory responses are similar, but not identical. More stringent prevention and management strategies for glycemic control in diabetic patients are strongly advocated.

## Data Availability

The datasets presented in this study can be found in online repositories. The names of the repository/repositories and accession number(s) can be found below: PRJNA1452658 (Bioproject, NCBI).

## Supplementary material

Supplementary Table 1 PCR primer sequences for this study.
