# Supplementary figures and images for: Hyperglycemia and systemic inflammation differentially shape immune dysregulation, tissue destruction, and microbiota in experimental periodontitis and peri-implantitis in diabetic mice
Source: Front Immunol. 2026 Jun 24;17:1847456. doi: 10.3389/fimmu.2026.1847456 (PMC13341294; doi:10.3389/fimmu.2026.1847456)

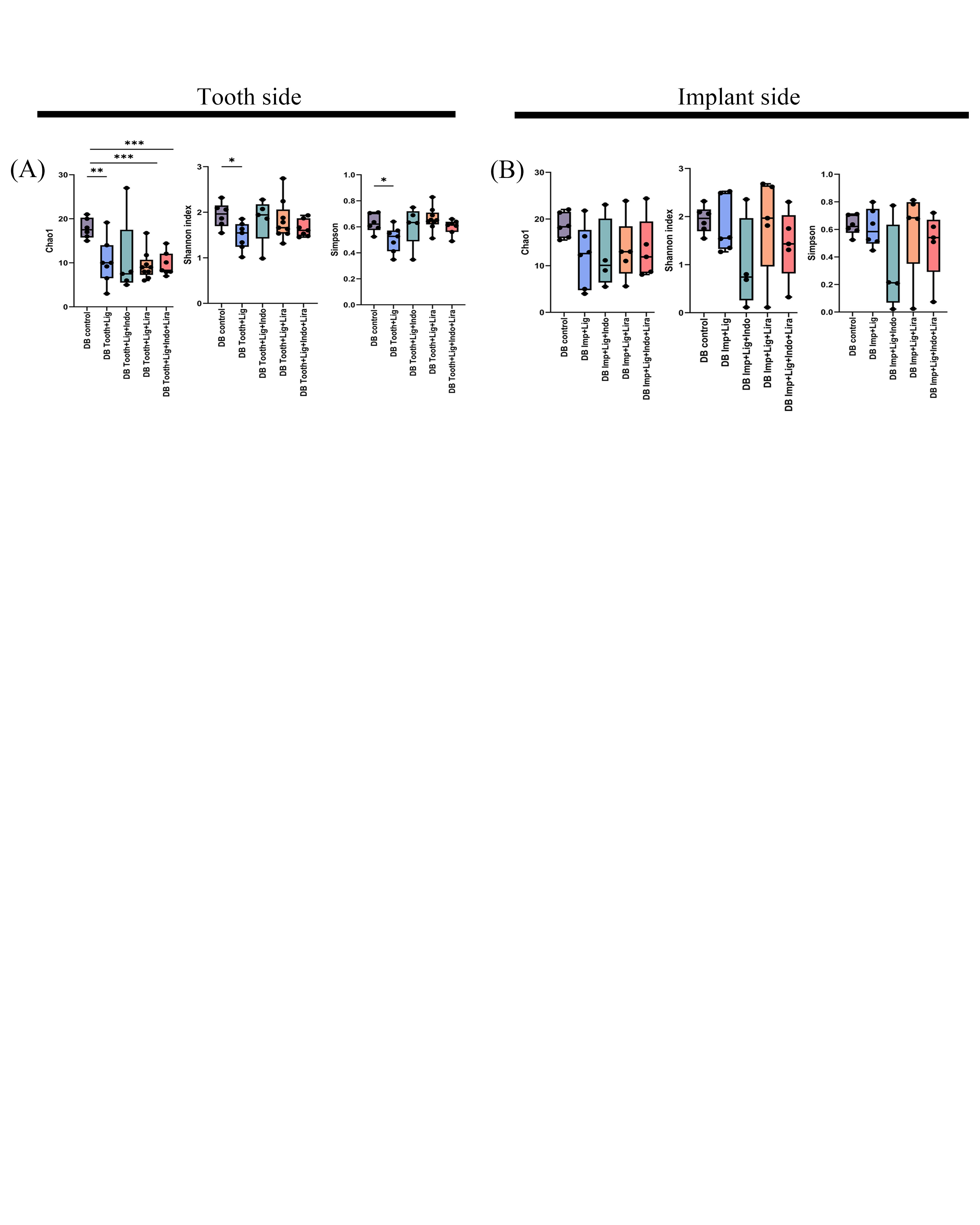

Supplement: Supplementary Table 1 — PCR primer sequences for this study. [file Image1.jpeg]
