# Supplementary material for: Hyperglycemia and systemic inflammation differentially shape immune dysregulation, tissue destruction, and microbiota in experimental periodontitis and peri-implantitis in diabetic mice
Source: Front Immunol. 2026 Jun 24;17:1847456. doi: 10.3389/fimmu.2026.1847456 (PMC13341294; doi:10.3389/fimmu.2026.1847456)
Supplement: Supplementary file 2 [file Table1.docx]

**Supplementary Table 1**

| *Gapdh* | Forward | CCTGGAGAAACCTGCCAAGTATG |
| --- | --- | --- |
|  | Reverse | TGTTGCTGTAGCCGTATTCATTGT |
| *Il10* | Forward | CTGAAGACCCTCAGGATGCG |
|  | Reverse | ACACCTTGGTCTTGGAGCTTAT |
| *Il1rn* | Forward | CGTTGGAAGGCAGTGGAAGA |
|  | Reverse | GGTTAGTATCCCAGATTCTGAAGGC |
| *Vegfa* | Forward | CTGCTGTAACGATGAAGCCCTG |
|  | Reverse | GCTGTAGGAAGCTCATCTCTCC |
| *Il1b* | Forward | CCTTGTGCAAGTGTCTGAAGC |
|  | Reverse | TCATCTTTTGGGGTCCGTCAAC |
| *Tnfa* | Forward | GGTGCCTATGTCTCAGCCTCTT |
|  | Reverse | GCCATAGAACTGATGAGAGGGAG |
| *Il17a* | Forward | CAGACTACCTCAACCGTTCCAC |
|  | Reverse | TCCAGCTTTCCCTCCGCATTGA |
| *Rage* | Forward | GCCACTGGAATTGTCGATGAGG |
|  | Reverse | GCTGTCAGTTCAGAGGCAGGAT |
| *Tnfsf11 (Rankl)* | Forward | CATGTGCCACTGAGAACCTTGAA |
|  | Reverse | CAGGTCCCAGCGCAATGTAAC |
| *Tnfrsf11b (Opg)* | Forward | AGCAGGAGTGCAACCGCACC |
|  | Reverse | TTCCAGCTTGCACCACGCCG |

**Table S1**: Primer sequences used for quantitative RT-PCR.
